# Red elemental selenium nanoparticles mediated substantial variations in growth, tissue differentiation, metabolism, gene transcription, epigenetic cytosine DNA methylation, and callogenesis in bittermelon (*Momordica charantia*); an *in vitro* experiment

**DOI:** 10.1371/journal.pone.0235556

**Published:** 2020-07-02

**Authors:** Sara Rajaee Behbahani, Alireza Iranbakhsh, Mostafa Ebadi, Ahmad Majd, Zahra Oraghi Ardebili

**Affiliations:** 1 Department of Biology, Science and Research Branch, Islamic Azad University, Tehran, Iran; 2 Department of Biology, Damghan Branch, Islamic Azad University, Damghan, Iran; 3 Department of Biology, North Tehran Branch, Islamic Azad University, Tehran, Iran; 4 Department of Biology, Garmsar Branch, Islamic Azad University, Garmsar, Iran; National University of Kaohsiung, TAIWAN

## Abstract

To gain a better insight into the selenium nanoparticle (nSe) benefits/toxicity, this experiment was carried out to address the behavior of bitter melon seedlings to nSe (0, 1, 4, 10, 30, and 50 mgL^-1^) or bulk form (selenate). Low doses of nSe increased biomass accumulation, while concentrations of 10 mgL^-1^ and above were associated with stem bending, impaired root meristem, and severe toxicity. Responses to nSe were distinct from that of bulk in that the nano-type exhibited a higher efficiency to stimulate growth and organogenesis than the bulk. The bulk form displayed higher phytotoxicity than the nano-type counterpart. According to the MSAP-based analysis, nSe mediated substantial variation in DNA cytosine methylation, reflecting the epigenetic modification. By increasing the concentration of nSe, the expression of the *WRKY1* transcription factor linearly up-regulated (mean = 7.9-fold). Transcriptions of phenylalanine ammonia-lyase (*PAL*) and 4-Coumarate: CoA-ligase (*4CL*) genes were also induced. The nSe treatments at low concentrations enhanced the activity of leaf nitrate reductase (mean = 52%) in contrast with the treatment at toxic concentrations. The toxic concentration of nSe increased leaf proline concentration by 80%. The nSe supplement also stimulated the activities of peroxidase (mean = 35%) and catalase (mean = 10%) enzymes. The nSe-treated seedlings exhibited higher PAL activity (mean = 39%) and soluble phenols (mean = 50%). The nSe toxicity was associated with a disrupted differentiation of xylem conducting tissue. The callus formation and performance of the explants originated from the nSe-treated seedlings had a different trend than that of the control. This experiment provides new insights into the nSe-associated advantage/ cytotoxicity and further highlights the necessity of designing convincing studies to introduce novel methods for plant cell/tissue cultures and agriculture.

## Introduction

Nanoscience and nanotechnology as multidisciplinary fields provide breakthrough functions in plant and agriculture sciences [1]. Due to the unique and outstanding physicochemical properties of nanoparticles, the nano-compounds induce differential responses in biological systems compared to the bulk substances [1–3]. In this regard, the synthetic method, concentration, physicochemical traits, and biological system species are major determining factors contributing to the potential advantages or risks of biological applications of nanoproducts [1–3].

Selenium (Se) is a metalloid element considered to be a vital essential micronutrient in humans and many other living organisms [4, 5]. However, its essentiality and function for plant species remain elusive. Se-associated changes in plant growth, biochemistry, and productivity depend on several determining factors, including Se type, experimental procedures, developmental stage, and plant species. In a narrow range of doses, application of Se may increase growth rate [6, 7], promote nutritional status [6], enhance photosynthesis efficiency [8], reprogram nuclear transcription profile [6–9], induce antioxidant system [6, 7, 10], stimulate both primary and secondary metabolism [6, 8], modify hormonal balances [5, 6], and improve plant acclimation to unfavorable environmental factors [4, 8]. On the other hand, several lines of evidence highlighted risks associated with high concentrations of Se [6, 7, 11, 12]. Moreover, recent reports point out the plant cell may differentially respond to the nano-based materials relative to the bulk counterparts [6, 7, 13]. The elemental red Se nano-substance (nSe) exhibits efficient antimicrobial properties, great bioactivity, considerable antioxidant capacity, and anti-proliferative effects [14]. The application of nSe in a dose-dependent manner associated with changes in growth, biochemistry, metabolism, and molecular program in diverse plant species, including *Triticum aestivum* [9], *Brassica juncea* [8], *Melissa officinalis* [6], sorghum [4], and peppermint [12]. Aside from the potential advantages of Se, some researchers have warned of the potential risks of phytotoxicity and environmental pollution [6–8, 11, 12]. Moreover, comparative convincing experiments on the interplay among bulk Se, nSe, and plant cells are rare and should be further investigated. In this regard, ongoing experiments, particularly under *in vitro* conditions, provide a great opportunity to fill the knowledge gaps in this area.

Adaptation or acclimation to diverse environmental factors is mediated through the transcriptional regulation of genes. In this regard, plant cells respond to both internal and external stimuli through the orchestrated signal perception and transduction processes with the contribution of various components among which transcription factors, phytohormones, ion transporters, kinases, and Ca^+2^ are the most important [15]. A transcription factor protein can be transcriptionally involved in the regulation of a cluster of downstream genes, thereby reprograming plant growth and biochemistry [15]. Hence, transcription factors are good target genes for illustrating the contributed mechanisms.

The superfamily of WRKYs directly or indirectly participate in up/down-regulation of various physiological processes and target defense-related genes during plant adaptation to stress limiting factors [16, 17]. Considering the involvement of the WRKY1 transcription factor in the modulation of developmental processes and stress-related genes [17, 18], we selected *WRKY1* as a target gene in this experiment. Taking secondary metabolism into account, the nSe-mediated differences in transcription of phenylalanine ammonia-lyase (*PAL*) and 4-Coumarate: CoA-ligase (*4CL*) were also monitored. Moreover, key steps of productions of major secondary metabolites with a phenolic structure are mediated through the catalytic actions of PAL and 4CL. Furthermore, salicylic acid is an important signaling agent produced with the catalytic action of PAL [19].

Regulation of the dynamic nature of chromatin is one of the important mechanisms necessary for modulation of gene expression. In this regard, the DNA methylation reaction plays a key modulatory role in the transition between heterochromatin and euchromatin natures in response to diverse internal and external cues. Hence, we attempted to explore the possible nSe-associated modifications in DNA methylation rate.

In both traditional and modern medicinal sciences, bitter melon (*Momordica charantia*), a member of Cucurbitaceae, is recognized as a pharmaceutically valuable herb. Bitter melon-derived medicines are widely utilized to remedy some of the most important and common human diseases, especially diabetes. The great biological activities of its extracts have been attributed to its complex phytochemistry resulting in the production of a multitude of valuable metabolites with terpenoid and phenolic structures [20].

Within this framework, we attempted to study the response of bitter melon seedlings to the bulk Se/nSe as a supplement in culture medium under sterile laboratory conditions. To gain a deeper insight into the potential benefits or phytotoxicity of nSe, various experimental evaluations were conducted to address nSe-associated changes in (I) plant morphology, growth, and anatomy, (II) primary and secondary metabolism, (III) antioxidant enzymes, (IV) expression of the *WRKY1* transcription factor, *PAL*, and *4Cl* genes, and (V) epigenetic cytosine DNA methylation. Another hypothesis was that explants' behavior of nSe-supplemented seedlings would be different from the control during the callogenesis process. Taking *in vitro* culture into account, the callogenesis performance of explants derived from the nSe-treated seedlings were also monitored in a supplementary experiment.

## Materials and methods

### Nano-product characteristics

**Fig 1:**
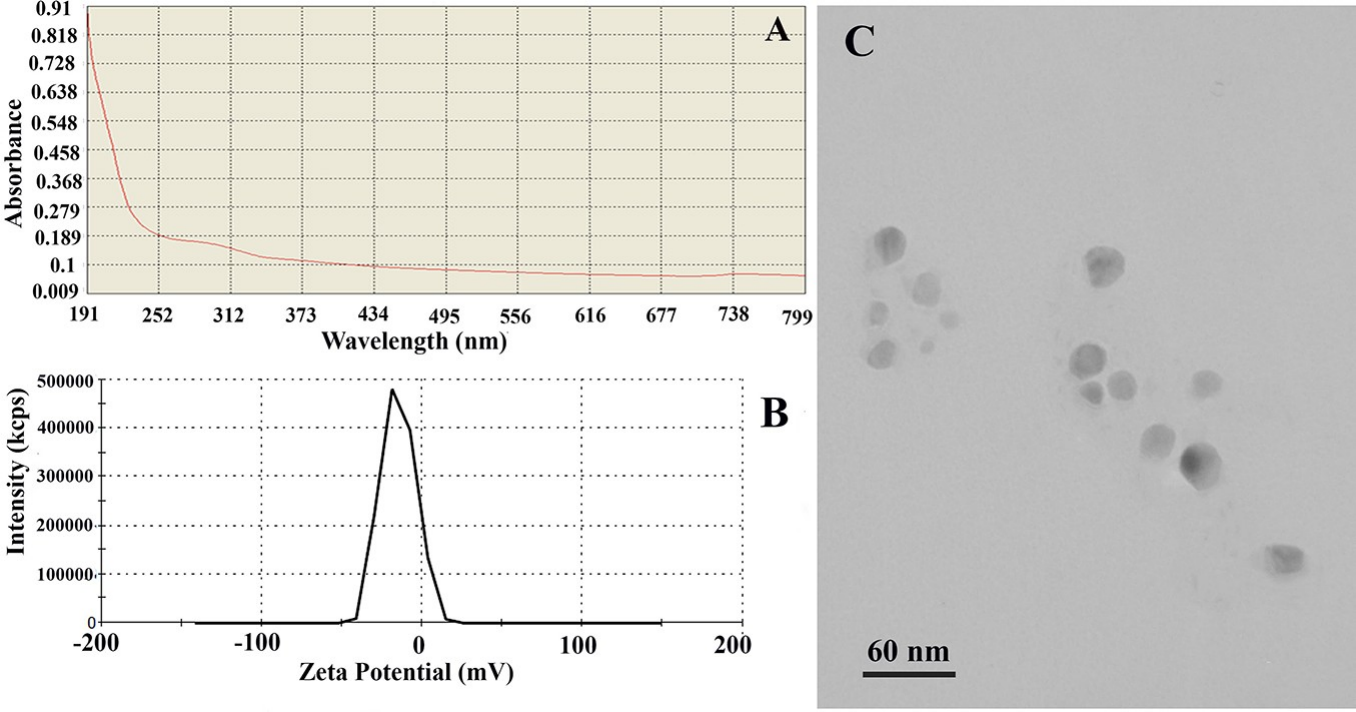
nSe physicochemical traits. (A) UV-Vis scan spectrum curves for 10 mgL^-1^. (B) Zeta potential spectrum. (C) Transmission electron microscopy (TEM) image.

Fig 1 exhibited the diverse physicochemical traits of nSe compound (CAS# 7446-08-4; APS, 10–45 nm; density, 3.89 gcm^−3^; high purity, 99.95%) supplied by the company (the NanoSany Corporation, Iranian Nanomaterials Pioneers Company, Mashhad City, Khorasan Province, Iran). Sodium Selenate (Na_2_SeO_4_; Molecular weight: 188.94) was applied as a bulk Se.

### Treatments and experimental conditions

The bitter melon seeds (PALEE, the East–West Seed International LTD, Nonthaburi 11110, Thailand) were disinfected in several consecutive stages using different compounds, including water containing Benomyl of 0.05% and 3 drops of liquid detergent for 4 min, Nystatin of 0.05% for 10 min, sodium hypochlorite of 1% for 10 min, and ethanol of 70% for 1 min. After each step, the seeds were washed with sterile distilled water. After that, the seeds were cultured in the hormone-free MS culture medium [21] containing various doses of nSe, including 0, 1, 4, 10, 30, and 50 mgL^-1^ or corresponding concentrations of the bulk form (selenate). The thirty-day old seedlings were subjected to further growth, physiological, anatomical, and molecular analysis.

### DNA methylation

Genomic DNA was extracted from the leaves using a particular kit (GeneAll, South Korea). In this experiment, the Methylation-Sensitive Amplification Polymorphism (MSAP) method previously explained by Guevara et al., [22] were used to compare the profile of DNA cytosine methylation between control and a group displayed nSe toxicity. Briefly, 500 ng of the genomic DNA was subjected for digestion by *Eco*RI/*Hpa*II or *Eco*RI/*Msp*I conditions at 37 °C for 2h. Next, five μl DNA fragments were exposed to adapters (Table 1) and T4 ligase (Fermentase, USA) with incubation at 22 °C for 1 h and subsequent incubation at 4 °C for 24 h. as a pre-amplification step, amplification of DNA fragments was performed using PCR (PeQlab; Germany) with primers (sequences are presented in Table 1). The next stage in the MSAP method was selective amplification stage in which amplifications were carried out using a thermocycler (PeQlab, Germany) and primers (sequences are displayed in Table 1). The amplified DNA fragments were undergone the polyacrylamide gel electrophoresis. Various software (R, Darwin, GeneALex, Past, and GraphPad) was applied. Data was finally interpreted with the MSAP package to detect non-methylated loci (NML) and methylation-susceptible loci (MSL).

**Table 1:** The sequences of the primers and adapters applied for the MSAP technique.

Primer/adapter	Sequences (5'-3')
Adapter MsHp-A1	GACGATGAGTCTAGAA
Adapter MsHp-A2	CGTTCTAGACTCATC
Adapter *Eco*RI-A1	CTCGTAGACTGCGTACC
Adapter *Eco*RI-A2	AATTGGTACGCAGTCTAC
PCR Primer *MsHp*-pre	GATGAGTCTAGAACGGT
PCR Primer *Eco*RI-pre	GACTGCGTACCAATTCA
PCR Primer *Eco*RI-ACT	GACTGCGTACCAATTCACT
PCR Primer *Eco*RI-AAG	GACTGCGTACCAATTCAAG
PCR Primer *MsHp*-TAC	GATGAGTCTAGAACGGTAC
PCR Primer *MsHp*-TC	GATGAGTCTAGAACGGTC

### Transcription of target genes

In this experiment, the nSe-associated variations in the expressions of target genes were addressed in leaves (before RNA extraction, leaves were kept in −80 °C). RNA was purified from leaves that were well-grounded in liquid nitrogen using DEPC Water (Bio Basic, Canada), triazole (GeneAll Biotechnology Co, South Korea), and Dnase I (Fermentase, USA). Then, the accuracy of RNA extraction was verified using a Nanodrop (Thermo Scientific^™^NanoDrop Model 2000c). Next, the synthesis of complementary DNA (cDNA) was carried out using a thermocycler (PEQLAB, 96Grad). In Table 2, the designed forward and reverse sequences of primers for Phenylalanine Ammonia Lyase (*McPAL*)- (XM_022284778), 4-Coumaroyl CoA ligase (*Mc4CL*)- XM_022281848, transcription Factor *WRKY1* (NW_019104495), and Elongation factor (a housekeeping gene) are depicted. After that, the transcription rates of the target genes were estimated according to common SYBR green (GeneAll, South Korea) and the real-time quantitative PCR procedure (Applied Biosystems StepOne^™^ Real-Time PCR). The delta CT protocol was utilized to calculate the expression rates presented as a fold difference.

**Table 2:** The forward and reverse sequences of primers for Phenylalanine Ammonia Lyase (*McPAL*; XM_022284778), 4-Coumaroyl CoA ligase (*Mc4CL*; XM_022281848, transcription Factor *WRKY1* (NW_019104495), and Elongation factor (a housekeeping gene).

Primer name	Sequence (5'-3')	Tm	Amplicon (bp)
*PAL*-F	ATTGGGAAGCTCATGTTTGC	57	177
*PAL*-R	GGTGACGGGATTTGCTAAGA	57.6	
*WRKY1*-F	AGTGATGGCATAGTCCTCGAT	59	112
*WRKY1*-R	CCAGGAATATGCGAACTTAGCTT	59	
*4CL*-F	ACCTCTGTTCCATGTTTTCGGAT	61	142
*4CL*-R	AGCAACCGATATGTACGTGACC	61	
*EFa*-F	GAACCGTGCTTGCTCAACCTC	61	138
*EFa*-R	AGCCGCAACAACTATGATCACC	61	

### Preparation of enzyme extract

Enzyme extracts were prepared by homogenizing the liquid nitrogen-grounded tissues in the phosphate buffer (0.1 M; pH of 7.2) supplemented with ascorbate and Na_2_EDTA. Then, the supernatants were stored at −80 °C until further biochemical assessments. The nSe-associated changes in several important enzymes were monitored according to different experimental protocols. The nitrate reductase activity [23] in leaves, catalase activity [24] in roots, peroxidase activity [11] in roots, and PAL activity [25] in roots were measured.

### Proline and soluble phenols

The proline contents [26] and concentrations of soluble phenols [12] were also quantified in leaves.

### Histological experiment

The stem and roots were subjected to cross-section procedure, stained within several common steps (sodium hypochlorite for 15 min, acetic acid for 5 min, carmine for 15 min, and methylene blue (30 s), and seen by a light microscope and photographed.

### Callogenesis experiments

Different explants (leaf, stem, and meristem) derived from the seedlings grown in MS medium containing nSe of 1 mgL^-1^ were subjected to callogenesis experiment. The explants were cultured in MS medium containing hormones (0.1 mgL^-1^ 2,4-D, 0.5 mgL^-1^ NAA, and 0.5 mgL^-1^ BAP).

### Statistical analysis

The experimental design was completely randomized. All data were subjected to analysis of variance (ANOVA) using SPSS software. The mean values of three independent replications for each treatment group were submitted to variance analysis by the Duncan test at a level of 5% of probability.

## Results

### Physicochemical characteristics of nanoparticle

It is well established that the physicochemical properties of nano-compounds play a determining role in their interaction with biological systems [24]. As shown in Fig 1A, a good absorption peak around 190–370 nm (UV region) was detected in the scan spectrum curves of nSe displaying nano-nature. This absorption spectrum did not alter over time indicating the stability of the compound. It is important to note that the transparent solution with no deposition indicated the appropriate solubility of the nanoparticles. Besides, the zeta potential index (-14.9 mV) reflected the existence of a negative surface charge and consequently confirmed the colloidal stability of the nano-compound due to electrostatic forces (Fig 1B). Besides, the nSe size ranging from 10 to 30 nm, and the spherical morphology is exhibited in the TEM image (Fig 1C).

### Growth and morphology

**Fig 2:**
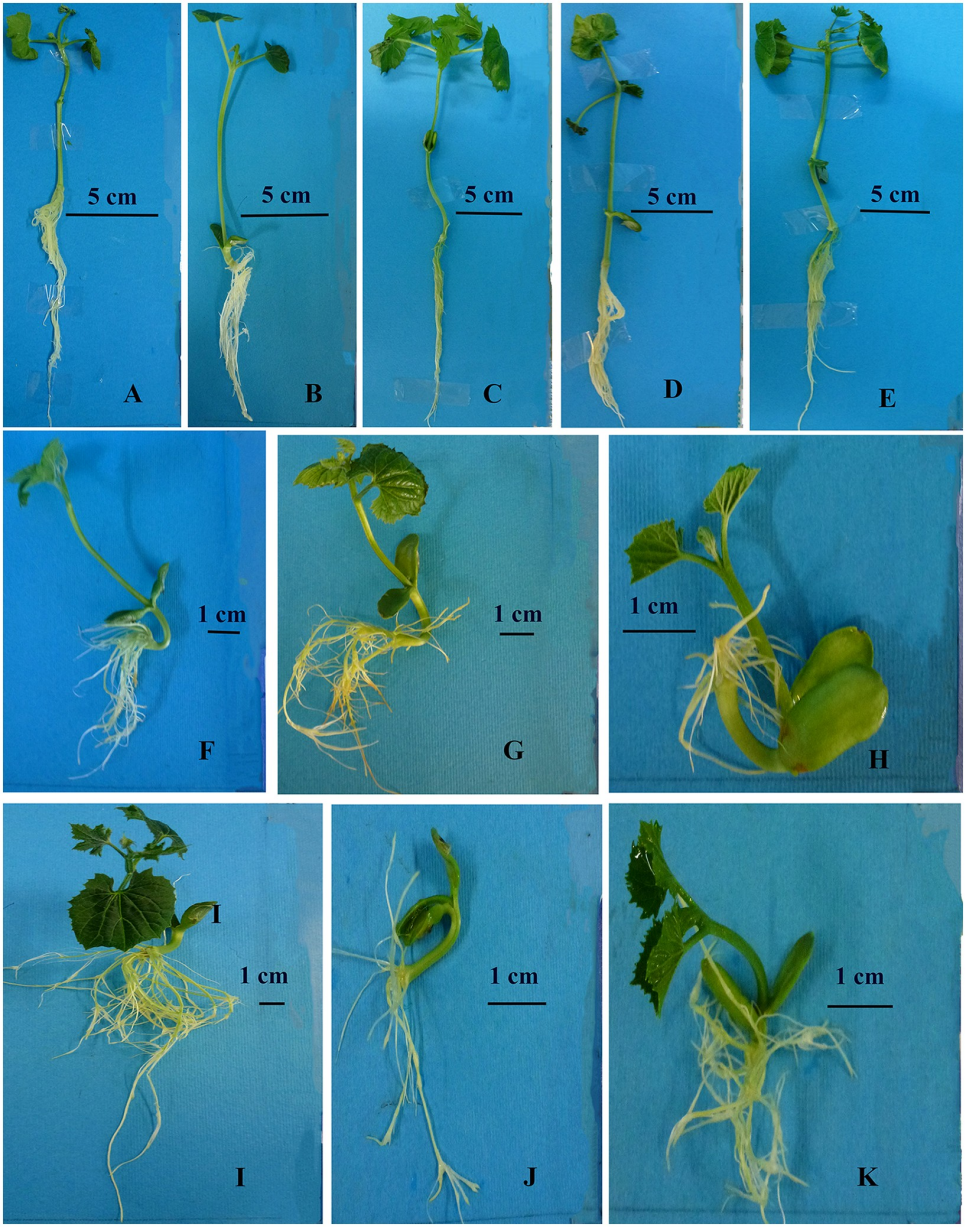
Differential morphology and growth in bitter melon seedlings cultured in media containing different concentrations of nSe or corresponding doses of its bulk counterpart (BSe). Stem bending, restriction in primary root development, and appearance of adventitious roots with a changed geotropism response are the main morphological symptoms of nSe toxicity. (A) Control. (B) BSe at 1 mgL^-1^. (C) nSe at 1 mgL^-1^. (D) BSe at 4 mgL^-1^. (E) nSe at 4 mgL^-1^. (F) BSe at 10 mgL^-1^. (G) nSe at 10 mgL^-1^. (H) BSe at 30 mgL^-1^. (I) nSe at 30 mgL^-1^. (J) BSe at 50 mgL^-1^. (K) nSe at 50 mgL^-1^.

The supplementation of MS culture medium with BSe or nSe influenced growth and morphology in both roots and shoot in a dose-dependent manner (Fig 2A–2K). Concentrations of 10 mgL^-1^ and above resulted in stem bending and severe growth inhibition of the organs, especially the roots. In addition to stem bending, the high doses of BSe/nSe, especially the bulk form, was associated with restriction in primary root development and appearance of adventitious roots with a changed geotropism response (Fig 2F–2K).

**Fig 3:**
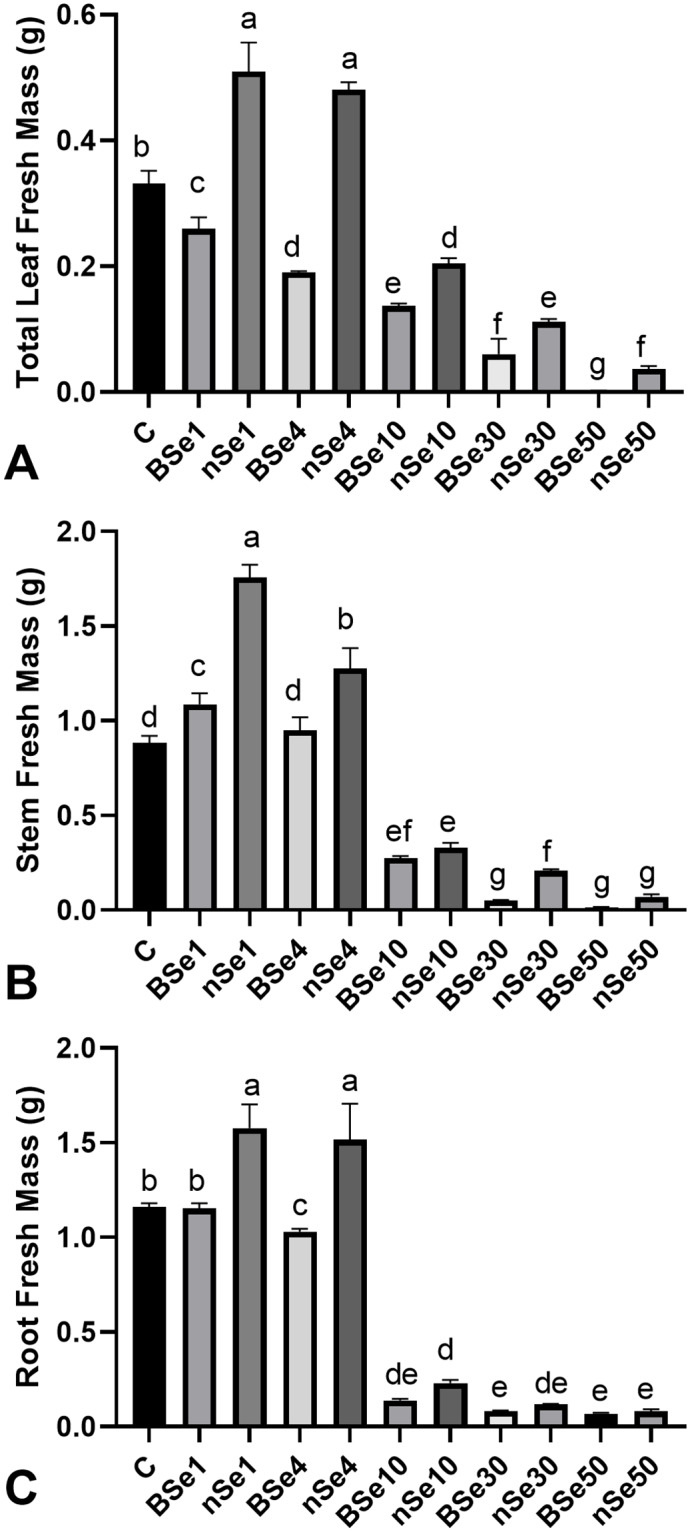
Variations in growth-related parameters following supplementation of culture medium with different doses of nSe (0, 1, 10, 30, and 50 mgL^-1^) or corresponding concentrations of BSe. Data are mean ± SD. (A) total leaf fresh mass. (B) stem fresh mass. (C) root fresh mass.

Moreover, nSe at concentrations of 1 or 4 mgL^-1^ significantly increased the total leaf fresh mass by an average of 50% when compared to the control (Fig 3A). However, the corresponding concentrations of the bulk counterpart only moderately decreased the total leaf fresh mass (mean = 31%) in comparison with the control (Fig 3A). The BSe1 (23.7%), nSe1 (about 2 folds), and nSe4 (45%) treatment groups had significantly higher stem fresh mass than the control (Fig 3B). The nSe treatments at 1 and 4 mgL^-1^ increased root fresh mass by 32.8% relative to the untreated control, while BSe at 4 mgL^-1^ only slightly (by 11%) reduced root fresh mass (Fig 3C). However, the other applied doses of nSe were highly toxic and inhibited root development (Fig 3C).

### DNA methylation

**Fig 4:**
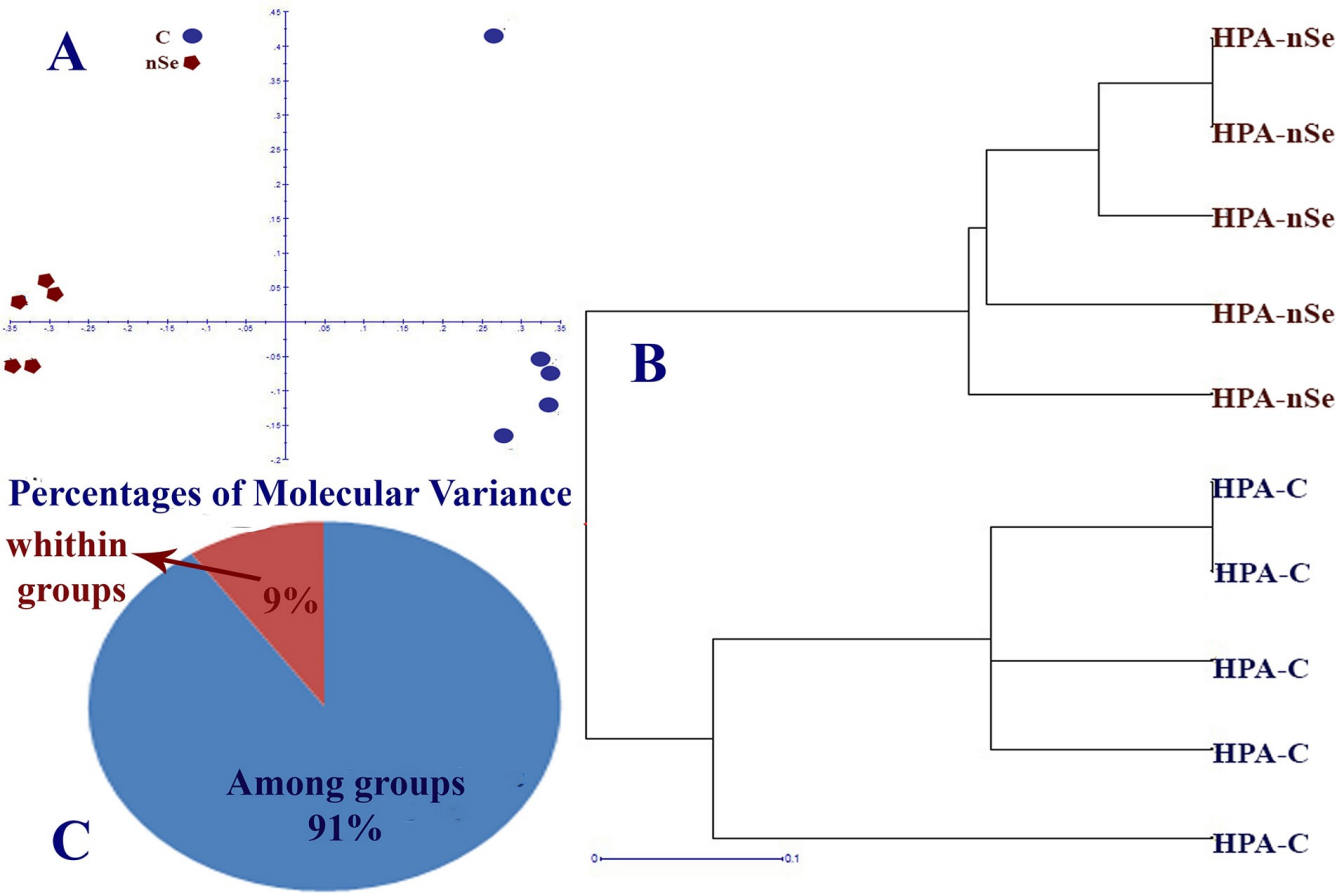
MSAP-based variation in DNA methylation patterns of bitter melon in response to the incorporation of nSe at 0 (control) and 30 mgL^-1^ into the MS culture medium. (A) Principal coordinates analysis (PCoA) plots of variation in DNA methylation under *Eco* RI/*Hpa* II conditions. (B) Clustering dendrogram based on DNA methylation variation between control and nSe treatment group; (C) Percentage of molecular variance within and among treatment groups.

**Fig 5:**
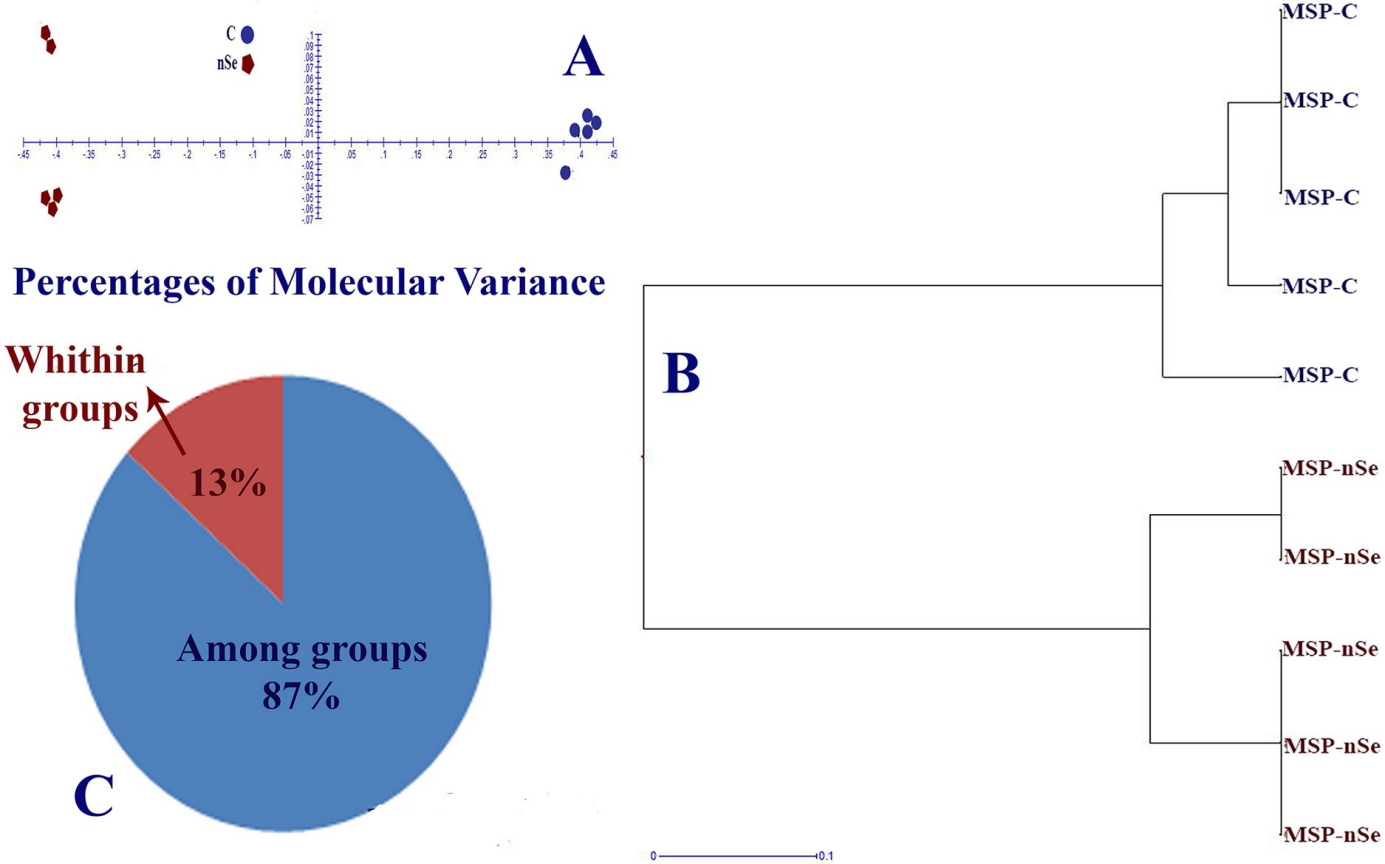
MSAP-based variation in DNA methylation patterns of bitter melon in response to the incorporation of nSe at 0 (control) and 30 mgL^-1^ into the MS culture medium. (A) Principal coordinates analysis (PCoA) plots of variation in DNA methylation under *Eco*RI/*Msp* I conditions. (B) Clustering dendrogram based on DNA methylation variation between control, and nSe treatment group. (C) Percentage of molecular variance within and among treatment groups.

**Fig 6:**
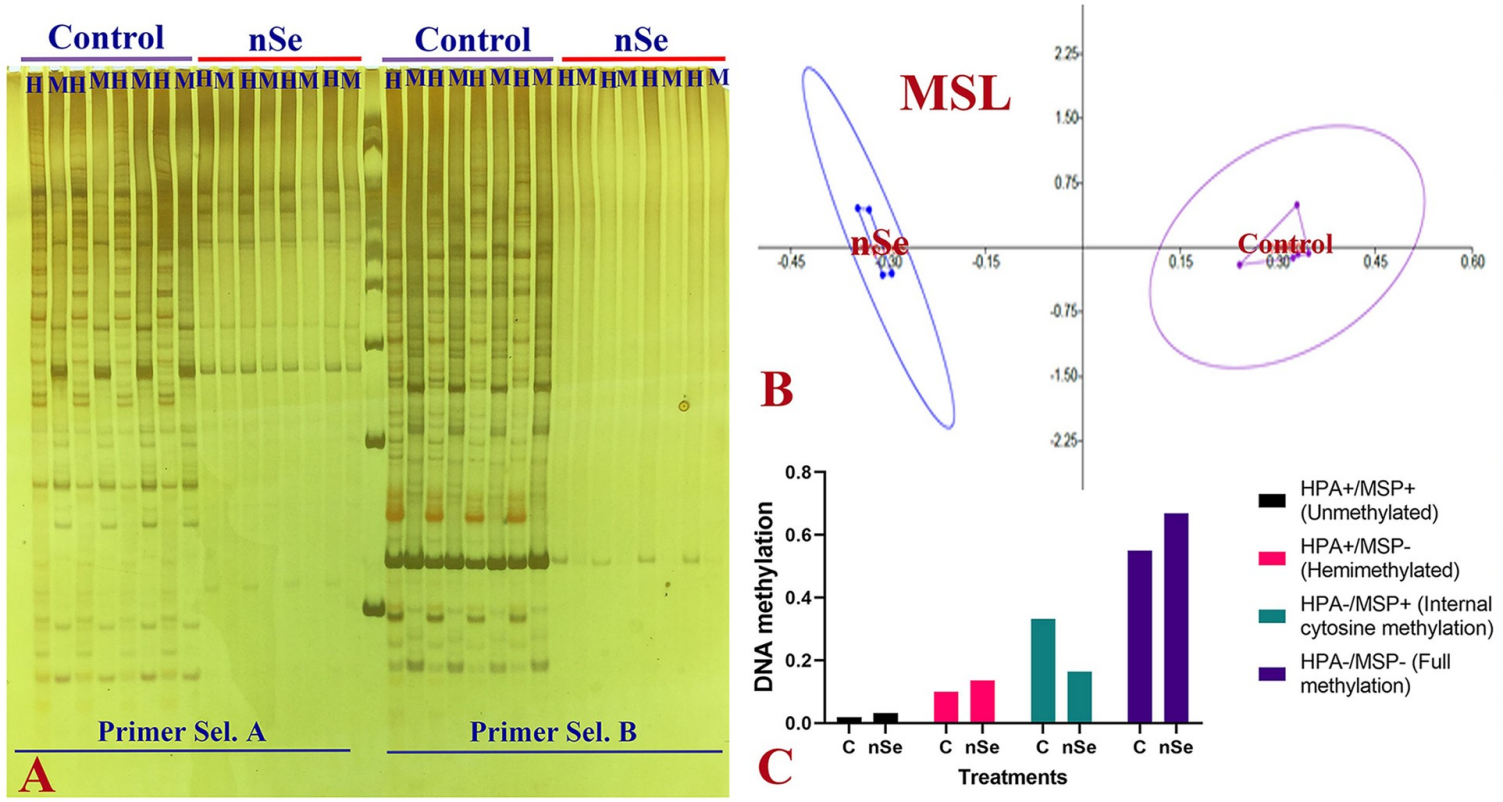
MSAP-based variation in DNA methylation patterns of *bitter melon* in response to the incorporation of nSe at 0 (control) and 30 mgL^-1^ into the MS culture medium. (A) A representative gel image exhibiting polymorphic fragments in control and nSe-treated group. (H) DNA digested under EcoRI/HpaII conditions. (M) DNA digested under EcoRI/MspI conditions. (B) PCoA plot showing variation in methylation-susceptible loci (MSL). (C) graph displaying differences in DNA methylation; HPA+ and MSP+ (1, 1) showing unmethylated; HPA+ and MSP- (1, 0) referring to hemimethylated; HPA- and MSP+ (0, 1) internal cytosine methylation; HPA- and MSP- (0, 0) exhibiting full methylation.

In this experiment, DNA cytosine methylation variations between control and the nSe treatment at a toxic dose were evaluated based on the MSAP method with the application of *Eco* RI/ *Hpa* II and *Eco* RI/ *Msp* I (*Hpa* II and *Msp* I are isoschizomers with differential methylation sensitivity). By comparing the monitored fragment profiles of *Eco* RI/*Msp* I and *Eco* RI/*Hpa* II, two kinds of polymorphisms can be identified. These polymorphisms are methylation-insensitive polymorphisms displaying polymorphic patterns among samples in response to *Eco* RI/*Msp* I and *Eco* RI/ *Hpa* II as well as methylation-sensitive polymorphisms showing the difference between *Eco* RI/ *Hpa* II and *Eco* RI/ *Msp* I profiles [22]. Comparison of MSAP fragment profiles of *Eco*RI/ *Ms* I and *Eco*RI/ *Hpa*II revealed methylation-sensitive and insensitive polymorphisms. Among 49 loci, the number of Methylation-Susceptible Loci (MSL) was 44, while the number of non-methylated Loci (NML) was 5. The principal coordinates analysis (PCoA) plots (Fig 4A) and dendrogram (Fig 4B) confirmed the variation in DNA methylation under *Eco* RI/*Hpa* II conditions. In this regard, the percentage of molecular variance within and among treatment groups was estimated to be 9% and 91%, respectively (Fig 4C). Likewise, PCoA plots (Fig 5A) and dendrogram (Fig 5B) exhibited differential DNA methylation patterns under *Eco* RI/*Msp* I conditions. The percentage of molecular variance within and among groups were 13% and 87%, respectively (Fig 5C). Variation in methylation-susceptible loci (MSL) between control and nSe-treated group indicated the epigenetic modification in response to nSe (Fig 6A–6C). Fig 6C showed variations in non-methylation, Hemi-methylation, internal cytosine methylation, and full methylation responses following the nSe treatment.

### Transcription of target genes (*WRKY1*, *PAL*, and *4CL*)

**Fig 7:**
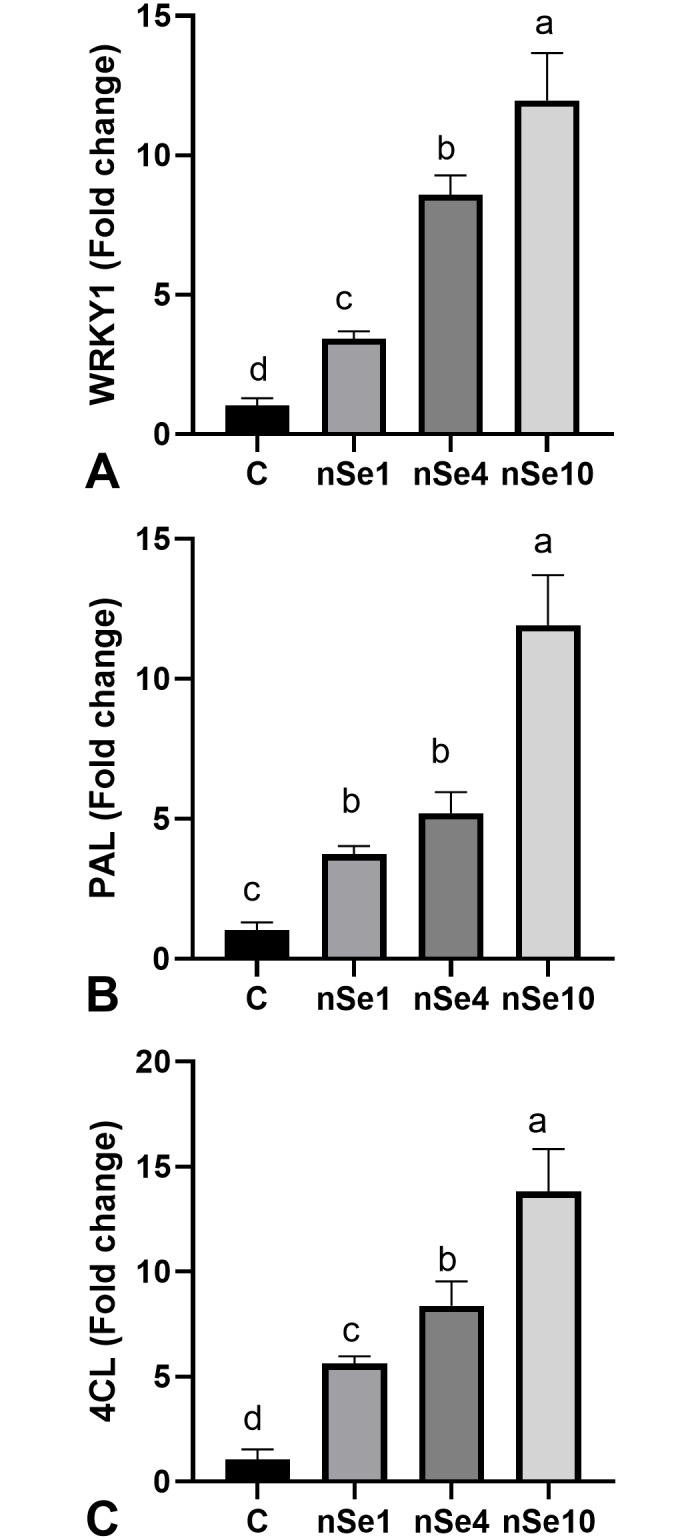
nSe-associated induction in the transcription of *WRKY1*, *PAL*, and *4CL*. Data are mean ± SD. (A) *WRKY1*. (B) *PAL*. (C) *4CL*.

By increasing concentrations of nSe, the expression of the *WRKY1* gene linearly up-regulated about 7.9 folds when compared to the untreated control samples (Fig 7A). In comparison to the control, the moderate upregulation (4.5 folds) in the expression of the *PAL* gene is caused by the nSe1 and nSe4 treatments (Fig 7B), while the drastic induction (11.9-fold) in the *PAL* transcription resulted from the nSe10 treatment (Fig 7B). With a similar trend, the expression of the *4CL* gene was significantly upregulated by an average of 9 folds relative to the control (Fig 7C).

### Physiological responses

**Fig 8:**
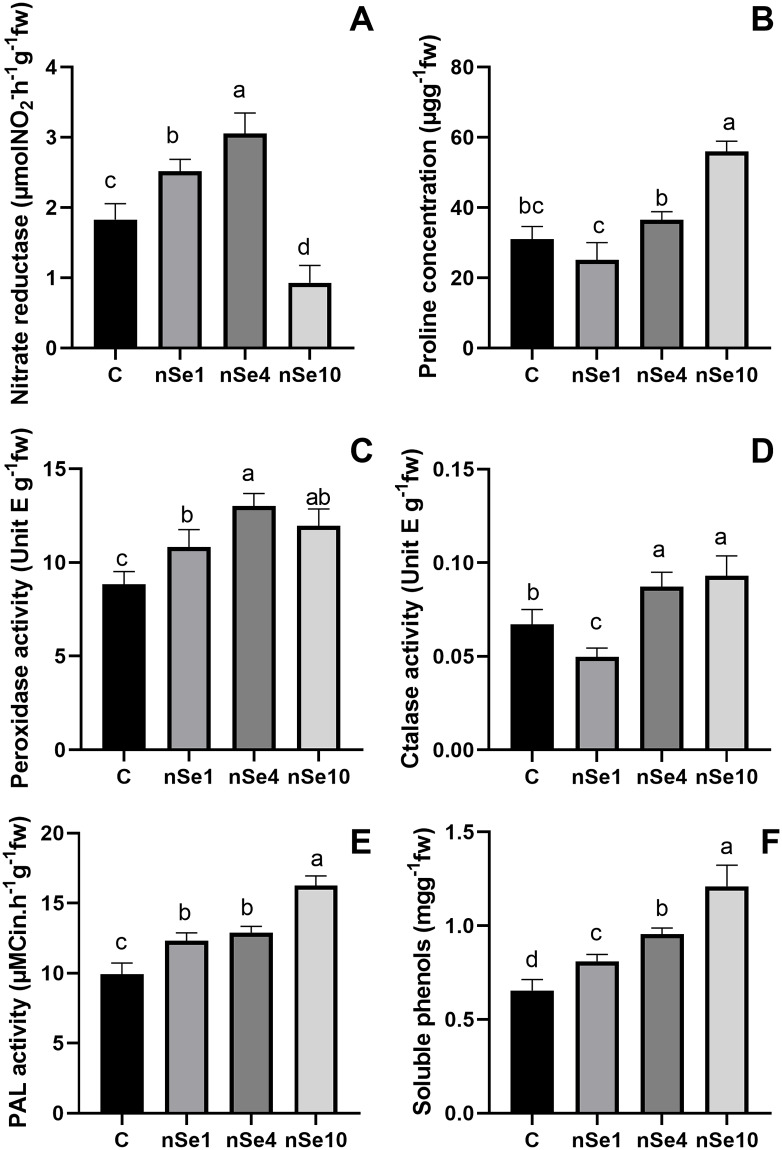
The nSe-mediated modifications in different physiological parameters. Data are mean ± SD. (A) leaf nitrate reductase. (B) leaf proline concentration. (C) root peroxidase activity. (D) root catalase activity. (E) root PAL activity. (F) total soluble phenols.

The nSe1 and nSe4 treatments significantly induced the activity of leaf nitrate reductase by an average of 52% when compared to the control (Fig 8A). In contrast, nSe at 10 mgL^-1^ led to a significant decrease in nitrate reductase activity in the leaves compared to the control (Fig 8A). The application of nSe10 caused a significant rise (80%) in leaf proline concentration (Fig 8B). However, the differences between nSe1, nSe4, and control groups were not statistically significant (Fig 8B). The presence of nSe moderately stimulated the root peroxidase activity by an average of 35% relative to the control (Fig 8C). Except for nSe1, the exposure to nSe significantly induced root catalase activity (Fig 8D). The nSe-treated seedlings exhibited significantly higher PAL activity in roots by an average of 39% over that of the control (Fig 8E). Also, the applied supplement significantly enhanced the leaf soluble phenols by a mean of 51% when compared to the control (Fig 8F).

### Stem and root anatomy

**Fig 9:**
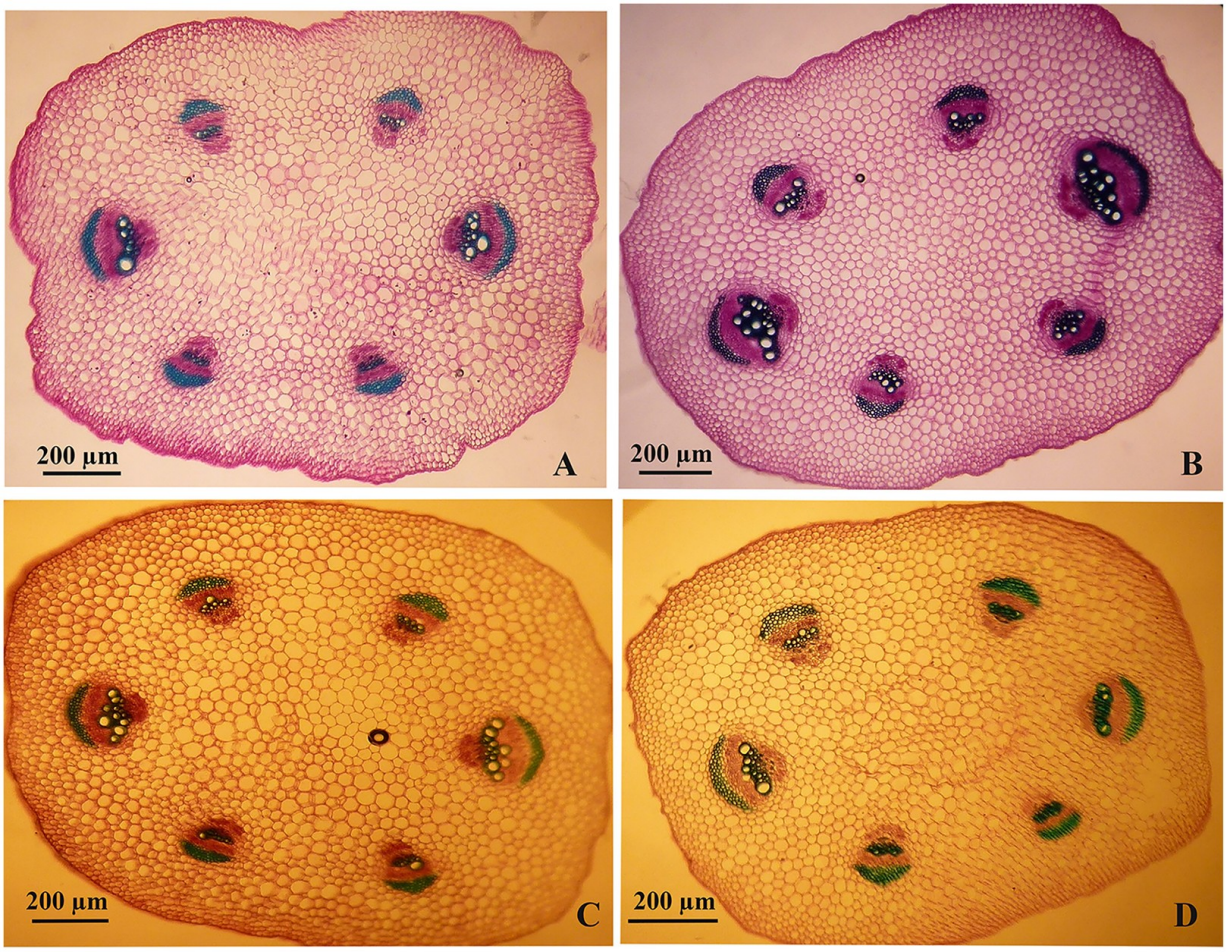
Anatomical variations in stem structure and differentiation of xylem conducting tissue in response to the supplementation of the MS culture medium with different concentrations of nSe. (A) Control. (B) 1 mgL^-1^. (C) 4 mgL^-1^. (D) 30 mgL^-1^.

**Fig 10:**
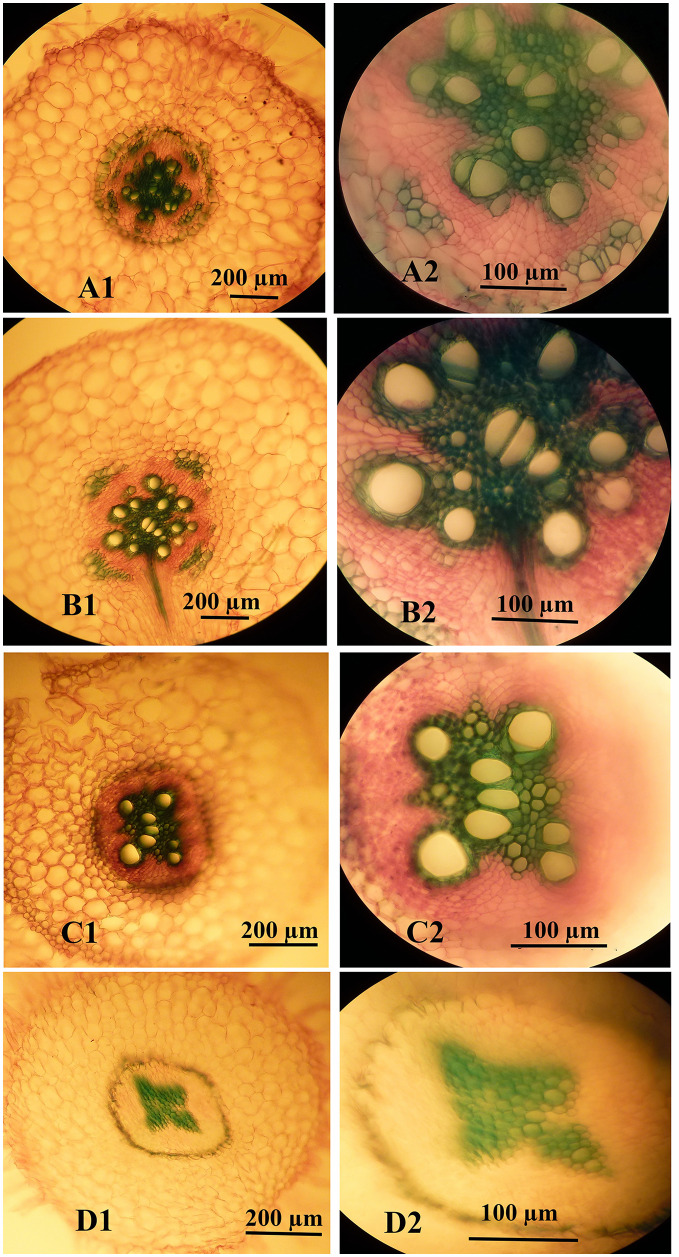
Anatomical variations in root structure and differentiation of xylem conducting tissue in response to the supplementation of the culture medium with different concentrations of nSe. (A1 and A2) Control. (B1 and B2) 1 mgL^-1^. (C1 and C2) 4 mgL^-1^. (D1 and D2) 30 mgL^-1^.

As highlighted above, the high concentrations of nSe were associated with impaired root development. Hence, we aimed to monitor possible variations in stem and root anatomy. At low doses (1 and 4 mgL^-1^, especially the former), the nSe supplementations improved the development of conducting vascular tissues, as indicated by increases in both the number and the diameter of differentiated metaxylem elements (Fig 9B and 9C). However, nSe treatment at a toxic dose (30 mgL^-1^) was associated with a delay in the differentiation of primary vascular tissues. Micro-measurement analysis revealed that nSe of 1 mgL^-1^ considerably increased the stem diameter, the length of the cortex layer, and the diameter of the central cylinder when compared to the control. On the other hand, nSe at 30 mgL^-1^ adversely influenced anatomical indices and decreased the stem diameter, the length of the cortex layer, and the central cylinder diameter. Likewise, nSe treatments at 1 mgL^-1^ considerably reinforced the root structure (Fig 10B1 and 10B2), while, the incorporations of nSe at 30 mgL^-1^ severely halted differentiations of xylem conducting tissue (Fig 10D1 and 10D2). Micro-measurement assessments confirmed that nSe treatments at 1 mgL^-1^ mediated increases in the development of primary and secondary tissues, the metaxylem diameter, the length of the cortex layer as well as the diameter of the central cylinder. In contrast, nSe at 30 mgL^-1^ inhibited differentiation of xylem conducting tissue which may be responsible for halted growth of seedlings.

### Callogenesis experiment

**Fig 11:**
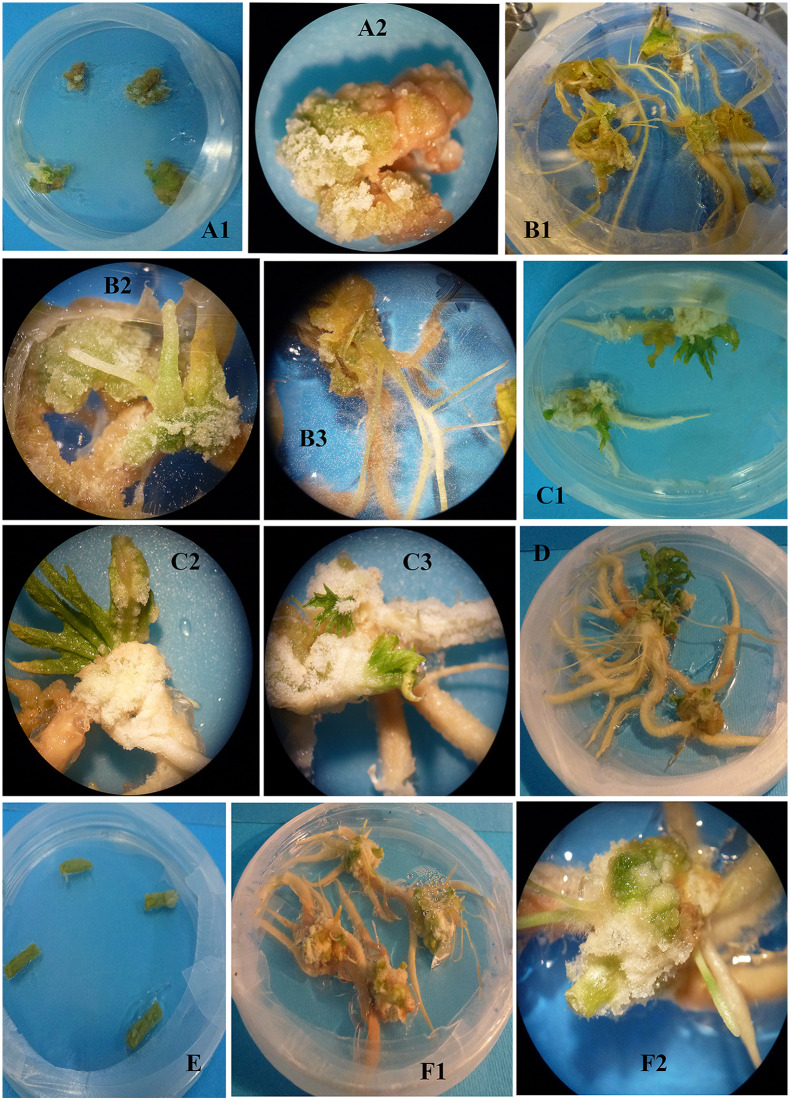
Comparative callogenesis or organogenesis potential of various explants (leaf, stem, and meristem) derived from the seedlings grown in MS medium containing nSe of 1 mgL^-1^. The explants were cultured in MS medium containing hormones (0.1 mgL^-1^ 2,4-D, 0.5 mgL^-1^ NAA, and 0.5 mgL^-1^ BAP). (A1 and A2) leaf explant derived from the control seedlings. (B1, B2, and B3) Leaf explant originated from the nSe-treated samples. (C1, C2, and C3) Meristem explant of control. (D) Meristem explant of nSe-treated samples. (E) stem explant of control. (F1 and F2) Stem explant derived from the nSe-treated plants.

The results showed that the callus formation and performance of the explants originated from the nSe-treated seedlings had a different trend than the control. Control leaf explants formed callus whereas callus formation from the corresponding explants of the nSe-treated plants was associated with root development and appearance of polar structures suspected to be somatic embryo (Fig 11A1, 11A2, 11B1, 11B2 and 11B3). When the apical meristems were used as explants, the organogenesis process in the control was different from that of the nSe-treated samples (Fig 11C1, 11C2, 11C3 and 11D). The root development in the nSe-treated meristem explants was significantly higher than the corresponding control. With a similar trend, stem explants of the control group did not undergo the dedifferentiation process, and callus or organogenesis was not recorded whereas stem explants originated from the nSe-treated seedlings were associated with the formation of adventitious root and embryo-like structures (Fig 11E, 11F1 and 11F2).

## Discussion

The incorporation of nSe as a supplement in the culture medium influenced morphology, growth, anatomy, and development in a manner dependent on the applied dose and the Se form. Responses to the nSe exposure were partly distinct from that of BSe, in line with the findings of Babajani et al., [6] in *Melissa officinalis* and Sotoodehnia-Korani et al., in *Capsicum annuum* [7]. The nano-type Se (nSe) exhibited a higher efficiency to stimulate plant growth and organogenesis than the bulk form. Moreover, the nano-form was less toxic than the selenate (bulk Se). This can be attributed to the differential physicochemical characteristics of nanomaterial (high surface/volume ratio) determining its uptake, reception, translocation, and interaction with biomolecules and cellular organelles, as well as signal transduction. Numerous studies have addressed the differential responses of various biological systems to nanomaterials compared to the bulk counterpart substances [6, 13]. Furthermore, researchers underlined several major determining factors, including the synthesis method [1–3], utilized concentration [7], application procedure [6], exposure time [9], physicochemical traits [1, 2, 3, 12], developmental stage [9, 12], and biological system species [13, 27] through which beneficial functions or the cytotoxicity of nanoproducts can be varied. In this regard, convincing evidence has been provided that the uptake kinetics of nSe and its subsequent metabolism in plant cells differ from the bulk counterpart [6, 7, 11]. The comparative experiment on the transport kinetics of nSe and selenite as bulk in wheat revealed a distinct involved mechanism [14]. Likely, the nSe uptake and translocation, as well as its interactions with biomolecules are distinct from the other forms of bulk Se. High concentrations of BSe/nSe restricted the development of both shoot and root organs. However, these toxic doses made no chlorosis and necrosis (two common noticeable toxicity symptoms). This can be considered for producers of ornamental plants and industrially exploited for the production of ornamental seedlings. Moreover, the high concentration of nSe was associated with stem bending, inhibition of primary root development, and appearance of adventitious roots with a changed geotropism response. The toxic dose of nSe inhibited differentiation of xylem tissue which may be considered as an underlying toxicity mechanism. These morphological and anatomical differences in response to nSe are clear indications of hormonal changes, especially ethylene and auxin as well as impairment in the apical meristems. Therefore, it is recommended to investigate nSe-mediated hormonal changes, especially at a molecular level, in future research. In line with our observations, nSe presence in the culture medium was reported to be associated with the abnormal stem apical meristem and repressed differentiation of xylem tissues in pepper seedlings, implying its cytotoxic role at a high concentration [7]. In *Astragalus fridae*, the supplementation of the culture medium with silica nanoparticles caused differential anatomy and tissue differentiation, especially conducting xylem tissue, in the stem, root, and leaf organs [13, 27]. However, there are knowledge gaps in the anatomical changes associated with biological applications of nanoparticles and more studies are, therefore, needed.

Exposure to Se may alter the intracellular status of reactive oxygen species (ROS) and reactive nitrogen species (RNS; especially nitric oxide (NO)). In response to Se, the root development was regulated through the antagonistic interplay between NO and ethylene hormone in *Arabidopsis* [28]. It could be stated that the nSe-triggered alterations in redox status and following signaling cascades may lead to changes in transcriptome, proteome, metabolome, and cellular differentiation. The redox-based regulation is considered a major mechanism that contributes to the remodeling of cellular transcriptional profile, chromatin architecture, and post-translational protein modifications [17].

According to the MSAP-based analysis, seedling exposure to the toxic dose mediated substantial variation in DNA cytosine methylation profile, reflecting the epigenetic response. The cytotoxicity caused by nSe triggered DNA hyper-methylations. Epigenetic modulation of the chromatin ultrastructure through DNA and histone modifications is a vital mechanism necessary for gene regulation at transcriptional levels in response to environmental stimuli. As is well known, DNA methylation is a determining factor towards chromatin architecture and gene accessibility to the transcriptional machinery. In line with our results, the cytotoxicity of nanoparticles was associated with DNA hypermethylation in *Capsicum annuum* [7]. In *Allium cepa*, DNA hyper-methylation was correlated with the cyto-genotoxicity of carbon nanotube [29]. It is worth mentioning that DNA cytosine methylation is a critical checkpoint control of cellular transcription program. Moreover, DNA hypermethylation epigenetically mediates the repression of gene expression. It appears that nSe perception and signal transduction associate with epigenetic modification, thereby altering growth, morphology, tissue differentiation, transcription program, and metabolism. Herein, the achieved findings highlight the necessity of tracing the epigenetic program during the cytotoxicity experiments of the nanoparticles.

Moreover, another supporting evidence presented in this experiment was that the differentiation of explants derived from the nSe-exposed seedlings was different from that of the corresponding control. These differences in callogenesis or organogenesis may indirectly reflect the nSe-associated endogenous differences, especially at epigenetics, phytohormones, and/or redox status. Considering stimulated root development and formations of embryo-like structures during the callogenesis or organogenesis experiment, the nSe-mediated changes in auxin, cytokinin, and abscisic acid hormones through NO/H_2_S signaling may be responsible for the differential callogenesis or organogenesis observed in this experiment; further studies are required to illustrate the exact features. Callogenesis or micropropagation processes can be modified by nanomaterials, like multiwalled carbon nanotubes [30] and copper oxide nanoparticles [31].

The nSe application led to linear upregulations in the *WRKY1* gene. With a similar trend, nSe treatment induced the transcriptions of *PAL* and *4CL* genes which are involved in secondary metabolism and production of a multitude of important secondary metabolites, especially salicylic acid (a key signaling bioagent). A WRKY1 transcription factor is the main constituent during signal transduction events followed by the regulations of downstream defensive genes conferring plant protection [10, 17]. For instance, the WRKY1 transcription factor contributes to the signaling route of salicylic acid (a vital hormone-like signaling agent) [32]. In grapevines, *WRKY1* overexpression was associated with the upregulation of jasmonic acid-related genes and subsequently enhanced resistance against stress [33]. This transcription factor is also implicated in the metabolic control of secondary metabolism [17]. In line with our results, the nSe application transcriptionally induced *WRKY1* and *bZIP* transcription factors in pepper [7]. Likewise, the exposure to nSe in a dose-dependent way accompanied by an alteration in the transcription pattern of heat shock factor A4A transcription factor [9]. Also, hydroxy phenylpyruvate reductase (*HPPR*) and rosmarinic acid synthase (*RAS*) genes were up-regulated in response to nSe treatment in Lemon balm [6]. Several reports highlighted Se-associated changes in endogenous phytohormones [5, 6, 7, 12, 34] as a crucial contributed mechanism. Likely, nSe-associated changes in hormones and intracellular redox levels following signal perception and transduction are responsible for the upcoming remodeled nuclear transcription program.

**Fig 12:**
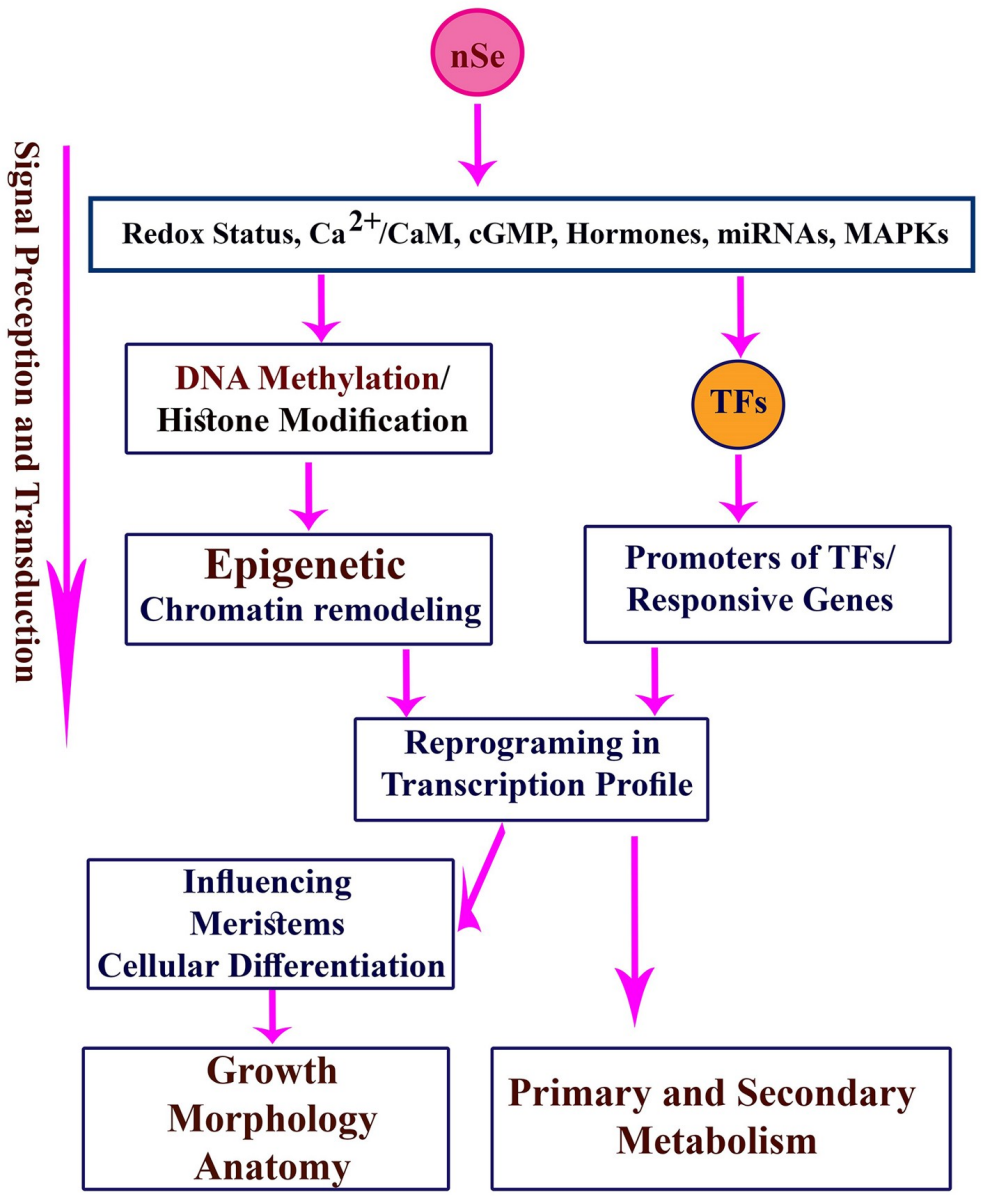
A schematic design on the possible nSe-associated mechanisms in plant cells. TFs: transcription factors.

The nSe application at low concentrations stimulated nitrate reductase activity, which can be considered an important indicator of nSe-mediated changes in primary metabolism, while high concentrations of nSe disrupt nitrate reductase activity and led to the proline accumulation. Consistent with our results, nSe-associated alterations in nitrate reductase activity [6, 7, 9, 12, 35] and proline concentrations [7, 12] have been recorded. Foliar application of Se improved nitrogen metabolism through stimulating activities of nitrate reductase and glutamate synthase in lettuce [35]. The Se-mediated changes in NO level are partly related to nitrate reductase activity [36]. The nSe supplementation modified activities of peroxidase and catalase (key components of enzymatic antioxidant machinery) in a concentration-dependent manner. The Se-mediated induction in antioxidant system is considered a critical mechanism that contributes to improving of plant protection against abiotic stress conditions [4–8, 10, 34]. The nSe compound also acted as an elicitor to stimulate secondary metabolism as it was indicated by induction in the activity of PAL enzyme (a vital enzyme in phenylpropanoid metabolism), up-regulation in expression of *4CL* and *PAL* genes, as well as an increase in concentrations of soluble phenols. These results are in line with several recent reports [4, 6, 7, 9, 12]. In Fig 12, a schematic model on the possible nSe-associated mechanisms is depicted.

## Conclusion

Taken together, based on the reported results in this study, it can be hypothesized that the *in vitro* application of nSe may be associated with novel functions in plant propagation and production of secondary metabolites. The results provide novel insights into the toxicity/advantage of nSe as an efficient elicitor in the culture medium. Responses to nSe exposure were partly distinct from that of BSe. The nano-type exhibited a higher efficiency to stimulate growth and organogenesis than the bulk. Moreover, in comparison to the corresponding bulk Se, nSe was less toxic, implying their differential interactions with biomolecules and cellular organelles, as well as signal transduction. Our results improve our knowledge of the possible ecotoxicological risks associated with nSe pollution. The phytotoxicity caused by high nSe dose triggered DNA hyper-methylations. Our results highlight this hypothesis that nSe can associate with epigenetic modification in DNA cytosine methylation, chromatin conformation, and cellular transcription program. Besides, this study underlines the importance of paying special attention to the role of transcription factors and epigenetic factors (DNA/histone-modifying machinery and miRNA) during signal perception and transduction of nanoparticles in the future toxicological studies, taking the current knowledge gaps into account. According to the present results, it can be concluded that nSe application at different developmental stages during plant cell and tissue culture procedures is a way by which cellular division, tissue differentiation, epigenetics, transcription profile, and metabolism can be manipulated. Future *in vitro* experiments can be designed to fill knowledge gaps, to clarify the mechanisms, and to elucidate the potential advantage or risks associated with biological applications of nSe.

## Supporting information

S1 Raw images (JPG)

S1 Data (CSV)

S2 Data (CSV)

## References

[1] Abbasi B, Iqbal J, Ahmad R, Zia L, Kanwal S, Mahmood T, et al Bioactivities of Geranium wallichianum Leaf Extracts Conjugated with Zinc Oxide Nanoparticles Biomolecules. 2020; 10(1): 38 10.3390/biom10010038 31888037 PMC7022592

[2] Iqbal J, Abbasi BA, Ahmad R, Mahmoodi M, Munir A, Zahra SA, et al Phytogenic Synthesis of Nickel Oxide Nanoparticles (NiO) Using Fresh Leaves Extract of Rhamnus triquetra (Wall) and Investigation of Its Multiple In Vitro Biological Potentials. Biomedicines. 2020; 8(5): p117 10.3390/biomedicines8050117 32408532 PMC7277790

[3] Abbasi BA, Iqbal J, Zahra SA, Shahbaz A, Kanwal S, Rabbani A, et al Bioinspired synthesis and activity characterization of iron oxide nanoparticles made using Rhamnus Triquetra leaf extract. 2020; Mater Res Express. 6(12): p1250e7. 10.1088/2053-1591/ab664d

[4] Djanaguiraman M, Belliraj N, Bossmann S, Prasad PV. High temperature stress alleviation by selenium nanoparticle treatment in grain sorghum. ACS Omega. 2018; 3(3): 2479–2491. 10.1021/acsomega.7b01934 31458542 PMC6641442

[5] Zahedi SM, Abdelrahman M, Hosseini MS, Hoveizeh NF, Tran L. Alleviation of the effect of salinity on growth and yield of strawberry by foliar spray of selenium-nanoparticles. Environ. Pollut. 2019; 253: 246–258. 10.1016/j.envpol.2019.04.078 31319241

[6] Babajani A, Iranbakhsh A, Ardebili ZO, Eslami B. Differential growth, nutrition, physiology, and gene expression in *Melissa officinalis* mediated by zinc oxide and elemental selenium nanoparticles. Environ Sci Poll Res. 2019; 26(24): 24430–24444. 10.1007/s11356-019-05676-z 31230234

[7] Sotoodehnia-Korani S, Iranbakhsh A, Ebadi M, Majd A, Ardebili ZO. Selenium nanoparticles induced variations in growth, morphology, anatomy, biochemistry, gene expression, and epigenetic DNA methylation in Capsicum annuum; an in vitro study. Environ Pollut. 2020; p114727 10.1016/j.envpol.2020.114727 32806441

[8] Handa N, Kohli SK, Sharma A, Thukral AK, Bhardwaj R, Abd_Allah EF. et al Selenium modulates dynamics of antioxidative defence expression, photosynthetic attributes and secondary metabolites to mitigate chromium toxicity in *Brassica juncea* L plants. Environ Exp Bot. 2019; 161: 180–192. 10.1016/j.envexpbot.2018.11.009

[9] Safari M, Ardebili ZO, Iranbakhsh A. Selenium nano-particle induced alterations in expression patterns of heat shock factor A4A (HSFA4A), and high molecular weight glutenin subunit 1Bx (Glu-1Bx) and enhanced nitrate reductase activity in wheat (Triticum aestivum L). Acta Physiol Plant. 2018; 40(6): p117 10.1007/s11738-018-2694-8

[10] Du C, Zhao P, Zhang H, Li N, Zheng L, Wang Y. The Reaumuria trigyna transcription factor RtWRKY1 confers tolerance to salt stress in transgenic Arabidopsis. J Plant Physiol. 2017; 215: 48–58. 10.1016/j.jplph.2017.05.002 28527975

[11] Babajani A, Iranbakhsh A, Ardebili ZO, Eslami B. Seed priming with non-thermal plasma modified plant reactions to selenium or zinc oxide nanoparticles: cold plasma as a novel emerging tool for plant science. Plasma Chem Plasma Process. 2019; 39(1): 21–34. 10.1007/s11090-018-9934-y

[12] Nazerieh H, Ardebili ZO, Iranbakhsh A. Potential benefits and toxicity of nanoselenium and nitric oxide in peppermint. Acta Agric Slov. 2018; 111(2): 357–368. 10.14720/aas.2018.111.2.11

[13] Moghanloo M, Iranbakhsh A, Ebadi M, Satari TN, Ardebili ZO. Seed priming with cold plasma and supplementation of culture medium with silicon nanoparticle modified growth, physiology, and anatomy in *Astragalus fridae* as an endangered species. Acta Physiol Plant. 2019; 41(4): 54 10.1007/s11738-019-2846-5

[14] Hu T, Li H, Li J, Zhao G, Wu W, Liu L. et al Absorption and bio-transformation of Selenium nanoparticles by wheat seedlings (*Triticum aestivum* L). Front Plant Sci. 2018 9: 597 10.3389/fpls.2018.00597 29868060 PMC5960721

[15] Khan SA, Li MZ, Wang SM, Yin HJ. Revisiting the role of plant transcription factors in the battle against abiotic stress. Int J Mol Sci. 2018; 19(6): 1634 10.3390/ijms19061634 29857524 PMC6032162

[16] Song H, Sun W, Yang G, Sun J. WRKY transcription factors in legumes. BMC Plant Biol. 2018; 18(1): p243 10.1186/s12870-018-1467-2 30332991 PMC6192229

[17] Iranbakhsh A, Oraghi Ardebili Z, Molaei H, Oraghi Ardebili N, Amini M. Cold Plasma Up-Regulated Expressions of WRKY1 Transcription Factor and Genes Involved in Biosynthesis of Cannabinoids in Hemp (*Cannabis sativa* L). Plasma Chem Plasma Process. 2020; 40: 527–537. 10.1007/s11090-020-10058-2

[18] Li C, He X, Luo X, Xu L, Liu L, Min L, et al Cotton WRKY1 mediates the plant defense-to-development transition during infection of cotton by *Verticillium dahliae* by activating JASMONATE ZIM-DOMAIN1 expression. Plant Physiol. 2014; 166(4): 2179–2194. 10.1104/pp.114.246694 25301887 PMC4256851

[19] Sheteiwy MS, An J, Yin M, Jia X, Guan Y, He F, et al Cold plasma treatment and exogenous salicylic acid priming enhances salinity tolerance of *Oryza sativa* seedlings. Protoplasma. 2019; 256(1): 79–99. 10.1007/s00709-018-1279-0 29984388

[20] Seddighinia FS, Iranbakhsh A, Ardebili ZO, Satari TN, Soleimanpour S. Seed priming with cold plasma and multi-walled carbon nanotubes modified growth, tissue differentiation, anatomy, and yield in bitter melon (*Momordica charantia*). J Plant Growth Regul. 2020; 39: 87–98. 10.1007/s00344-019-09965-2

[21] Murashige T, Skoog F. A revised medium for rapid growth and bio assays with tobacco tissue cultures. Physiol Plant. 1962; 15: 473–497.

[22] Guevara MÁ, de María N, Sáez-Laguna E, Vélez MD, Cervera MT, Cabezas JA. Analysis of DNA cytosine methylation patterns using methylation-sensitive amplification polymorphism (MSAP) In Plant Epigenetics (99–112) Humana Press, Boston, MA 2017; 10.1007/978-1-4899-7708-3_9 27770361

[23] Sym GJ. Optimisation of the in-vivo assay conditions for nitrate reductase in barley (*Hordeum vulgare* L cv Igri). J Sci Food Agric. 1984; 35: 725–30. 10.1002/jsfa.2740350703

[24] Salah SM, Yajing G, Dongdong C, Jie L, Aamir N, Qijuan H, et al Seed priming with polyethylene glycol regulating the physiological and molecular mechanism in rice (*Oryza sativa* L) under nano-ZnO stress. Sci Rep. 2015; 5: p14278. 10.1038/srep14278 26419216 PMC4588511

[25] Beaudoin-Eagan LD, Thorpe T. Tyrosine and phenylalanine ammonia lyase activities during shoot initiation in tobacco callus cultures. Plant Physiol. 1985; 78: 438–441. 10.1104/pp.78.3.438 16664262 PMC1064755

[26] Bates L, Waldren R, Teare I. Rapid determination of free proline for water-stress studies. Plant Soil; 1973; 39: 205–207. 10.1007/BF00018060

[27] Moghanloo M, Iranbakhsh A, Ebadi M, Ardebili ZO. Differential physiology and expression of phenylalanine ammonia lyase (PAL) and universal stress protein (USP) in the endangered species *Astragalus fridae* following seed priming with cold plasma and manipulation of culture medium with silica nanoparticles. 3Biotech. 2019; 9(7): 288 10.1007/s13205-019-1822-5 31297304 PMC6597672

[28] Feigl G, Horváth E, Molnár Á, Oláh D, Poór P, Kolbert Z. Ethylene-nitric oxide interplay during selenium-induced lateral root emergence in Arabidopsis. J Plant Growth Regul. 2019; 38: 1481–1488. 10.1007/s00344-019-09950-9

[29] Ghosh M, Bhadra S, Adegoke A, Bandyopadhyay M, Mukherjee A. MWCNT uptake in *Allium cepa* root cells induces cytotoxic and genotoxic responses and results in DNA hyper-methylation. Mutat Res. 2015; 774: 49–58. 10.1016/j.mrfmmm.2015.03.004 25829105

[30] Ghasempour M, Iranbakhsh A, Ebadi M, Ardebili ZO. Multi-walled carbon nanotubes improved growth, anatomy, physiology, secondary metabolism, and callus performance in *Catharanthus roseus*: an *in vitro* study. 3Biotech. 2019; 9(11): p404. 10.1007/s13205-019-1934-y 31681525 PMC6800878

[31] Anwaar S, Maqbool Q, Jabeen N, Nazar M, Abbas F, Nawaz B, et al The effect of green synthesized CuO nanoparticles on callogenesis and regeneration of *Oryza sativa* L. Front Plant Sci. 2016; 7: p1330. 10.3389/fpls.2016.01330 27630655 PMC5005396

[32] Bakshi M, Oelmüller R. WRKY transcription factors: Jack of many trades in plants. Plant Signal Behav. 2014; 9(2): pe27700. 10.4161/psb.27700 24492469 PMC4091213

[33] Marchive C, Léon C, Kappel C, Coutos-Thévenot P, Corio-Costet MF, Delrot S. et al Over-expression of VvWRKY1 in grapevines induces expression of jasmonic acid pathway-related genes and confers higher tolerance to the downy mildew. PLoS ONE. 2013; 8(1): pe54185. 10.1371/journal.pone.0054185 23342101 PMC3544825

[34] Soleymanzadeh R, Iranbakhsh A, Habibi G, Oraghi Ardebili Z. Selenium nanoparticle protected strawberry against salt stress through modifications in salicylic acid, ion homeostasis, antioxidant machinery, and photosynthesis performance. Acta Biol Cracov Bot. 2020; 10.24425/abcsb.2019.127751

[35] Bian ZH, Bo LE, Cheng RF, Yu WA, Tao LI, Yang QC. Selenium distribution and nitrate metabolism in hydroponic lettuce (*Lactuca sativa* L.): Effects of selenium forms and light spectra. J Integr Agric. 2020;19(1):133–44. 10.1016/S2095-3119(19)62775-9

[36] Lehotai N, Kolbert Z, Pető A, Feigl G, Ördög A, Kumar D, et al Selenite-induced hormonal and signalling mechanisms during root growth of *Arabidopsis thaliana* L. J Exp Bot. 2012; 63(15): 5677–5687. 10.1093/jxb/ers222 22988013

