# Supplementary figures and images for: Red elemental selenium nanoparticles mediated substantial variations in growth, tissue differentiation, metabolism, gene transcription, epigenetic cytosine DNA methylation, and callogenesis in bittermelon (Momordica charantia); an in vitro experiment
Source: PLoS One. 2020 Jul 2;15(7):e0235556. doi: 10.1371/journal.pone.0235556 (PMC7332037; doi:10.1371/journal.pone.0235556)

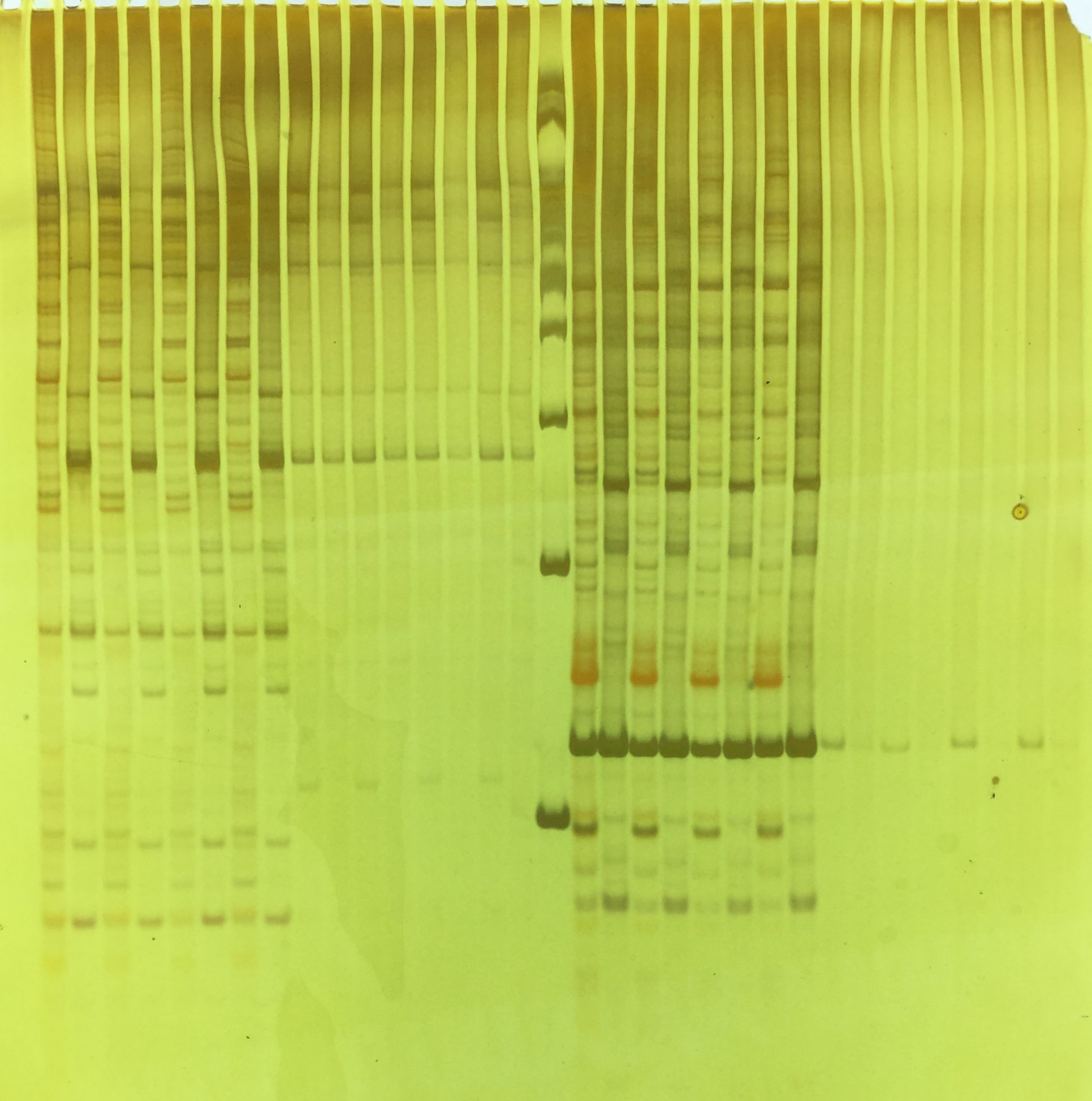

Supplement: S1 Raw images — (JPG) [file pone.0235556.s001.jpg]
